# Blood pressure monitoring in elderly migraineurs starting an anti-CGRP monoclonal antibody: a real-world prospective study

**DOI:** 10.1007/s10072-024-07567-9

**Published:** 2024-05-25

**Authors:** Davide Mascarella, Giorgia Andrini, Carlo Baraldi, Claudia Altamura, Valentina Favoni, Flavia Lo Castro, Giulia Pierangeli, Fabrizio Vernieri, Simona Guerzoni, Sabina Cevoli

**Affiliations:** 1 Department of Biomedical and Neuromotor Science DIBINEM, Alma Mater Studiorum Bologna, Bologna, Italy; 2 Digital and Predictive Medicine, Pharmacology and Clinical Metabolic Toxicology-Headache Center and Drug Abuse-Laboratory of Clinical Pharmacology and Pharmacogenomics, Department of Specialist Medicines, AOU Policlinico Di Modena, Modena, Italy; 3 Unit of Headache and Neurosonology, Department of Medicine and Surgery, Università Campus Bio-Medico Di Roma, Via Alvaro del Portillo, 21, 00128 Rome, Italy; 4 Fondazione Policlinico Universitario Campus Bio-Medico, Via Alvaro del Portillo, 200, 00128 Rome, Italy; 5 IRCCS Istituto Delle Scienze Neurologiche Di Bologna ISNB, Bologna, Italy

**Keywords:** Migraine, Anti-CGRP, CGRP, Blood pressure, Hypertension, Elderly, Erenumab, Galcanezumab, Fremanezumab, Cardiovascular safety

## Abstract

**Background:**

While monoclonal antibodies (mAbs) targeting the CGRP pathway have revolutionized migraine management due to their improved tolerance and adherence, concerns remain about their potential impact on blood pressure (BP), especially in older patients, due to CGRP-mediated vasodilation blockade. Given the growing use of these therapies in older populations, assessing their cardiovascular (CV) safety is of paramount importance.

**Methods:**

This multicentric observational prospective study focused on migraine sufferers aged ≥ 60 who began erenumab, galcanezumab, or fremanezumab for prevention. Baseline, three-month, and twelve-month BP measurements were collected. Changes in antihypertensive medication and "Newly or Worsened Hypertensive" patients (NWHP) were assessed.

**Results:**

Among 155 patients receiving anti-CGRP mAbs (40 Erenumab, 47 Galcanezumab, 68 Fremanezumab), 42.5% had hypertension history and 39% were on antihypertensive treatment. No significant systolic or diastolic BP changes occurred at any time point compared to baseline (all *p* > 0.05), with no differences between the three groups. After one year, 20/155 (12.9%) patients were considered NWHP; 11/20 had prior hypertension, and 5/11 adjusted antihypertensive therapy. Among 9/20 newly hypertensive patients, 5/9 had a single measurement above the normal threshold with no requirement for new pharmacological therapy. A higher baseline BP value was associated with increased BP (*p* = 0.002).

**Conclusions:**

The study concludes that treatment with anti-CGRP mAbs over one year does not significantly affect BP in patients aged ≥ 60, nor does it increase the incidence of hypertension compared to general population trends. Nonetheless, continuous monitoring and further long-term studies are necessary to fullya scertain the cardiovascular safety of these medications in the elderly.

## Introduction

Migraine is considered the second-highest cause of years lived with disability in the young-adult population [1]. While it is less prevalent among older individuals, the presence of comorbidities and the use of multiple medications can contribute to increased disability and economic burden [2, 3]. Particularly in older migraine patients, the co-occurrence of cardiovascular and cerebrovascular conditions may worsen outcomes and limit treatment options. The potential link between cardiovascular risk and migraine has been extensively discussed, yet a definitive clarification has not been reached. Whereas migraine may contribute to an elevated risk of cerebrovascular events, hypertension has been discussed as a potential risk factor for migraine chronification. [4–11]

Our understanding of migraine has shifted from considering it merely a blood vessel issue to a complex brain-centered disorder involving vascular structures. This is partly due to the discover and understanding of substances such as CGRP (calcitonin gene-related peptide), which play a crucial role in migraine pain development and also influence blood pressure (BP) regulation by dilating blood vessels, potentially protecting against heart disease and high blood pressure [12, 13].

However, its role as a beat-for-beat regulator of blood pressure is questioned by preclinical evidence: CGRP appears to have a greater vasodilatory effect when released from the trigeminal terminals than when released into the bloodstream where it shows a very short half-life. [14, 15] Consequently, CGRP-related vasodilation may be considered a response to a stimulus rather than a steady state BP regulator [16, 17].

The advent of monoclonal antibodies (mAbs) targeting CGRP (eptinezumab, fremanezumab, and galcanezumab) or its receptor (erenumab) has significantly improved both episodic and chronic migraine management offering minimal side effects. [18–27] Their widespread use, including among the elderly, offers a valuable treatment alternative for patients typically restricted by comorbidities. Consequently, there's a growing interest in the potential cardiovascular risks related to inhibiting CGRP-mediated vasodilation. [28, 29] Despite preclinical and clinical studies of anti-CGRP(r) mAbs designed to evaluate this theoretical risk did not demonstrate a link between these treatments and cardiovascular events, recent data suggest they may affect blood pressure, prompting a call for updates to their prescribing information [29, 30].

Except for post hoc analysis [31], there is no reported real-world prospective study in the literature specifically designed to evaluate the hemodynamic effect of selective blockade of CGRP or its receptor in the elderly population. Our study aims to evaluate the trend of systolic and diastolic BP in episodic or chronic migrainous patients aged over 60 years, treated with erenumab, fremanezumab, or galcanezumam, over a one-year follow-up period.

## Methods

### Study design

This was a multicenter, prospective, real-world, observational study. Patients from three tertiary headache centers (Bologna, Modena, and Campus Biomedico Rome) were prospectively recruited in order to evaluate mAbs preventive drugs erenumab, fremanezumab or galcanezumab effects on systolic and diastolic BP throughout 12 months follow-up.

Patients treated with erenumab started with 70 mg once every 4 weeks, with the option to titrate the dose to 140 mg if meaningful improvement was not achieved based on a shared decision between patients and physicians. Fremanezumab was prescribed 225 mg per dose monthly or 675 mg quarterly. Patients treated with galcanezumab received 240 mg as the loading dose, followed by 120 mg injections monthly.

BP measurements (mmHg) were performed throughout the treatment period at every follow-up visit. All measurement were performed in sitting position after a minimum rest period of 15 min with a digital sphygmomanometer. At first prescription demographic and clinical data, including age, sex, comorbidities (i.e., hypertension, dyslipidemia, and diabetes), age at migraine onset, and pharmacologic history were collected.

After treatment initiation, a follow-up visit was scheduled approximately after three months and after one year from the beginning of anti-CGRP treatment.

Patients with increased BP according to international blood pressure guidelines (16) at any time during the 12 months, were referred to the General Practitioner for close BP monitoring and evaluation of anti-hypertensive therapy start or adjustment.

### Patients’ eligibility criteria

All consecutive patients diagnosed with migraine referred to the centers between January 2019 till November 2021 who met the inclusion criteria were enrolled and followed up for at least 12 months. Inclusion criteria were: (i) diagnosis of migraine according to the International Classification of Headache Disorders-Third edition (ICHD-3), with ≥ 8 migraine days per month, who failed on ≥ 3 migraine preventives (discontinued because of lack of efficacy and/or tolerability reasons as self-reported by patients, or contraindicated) including at least a beta-blocker, anticonvulsant and antidepressant, according to the Italian Regulatory Agency indications for anti-CGRP mAbs reimbursement (ii) age ≥ 60 years, (iii) initiation of an anti-CGRP mAb (erenumab, fremanezumab or galcanezumab) after the baseline visit.

Exclusion criteria were: (i) Patients suffering from major cardiovascular/cerebrovascular conditions (i.e., previous ischemic stroke or transient ischemic attack, previous ischemic heart disease and myocardial infarction, previous deep vein thrombosis), according to the Italian regulatory agency. (ii) patients with ongoing diagnostic work-up for suspected secondary hypertension, according to the Italian regulatory agency; (iii) patients with uncontrolled hypertension despite poly-pharmacotherapy, according to the Italian regulatory agency; (iv) patients who started a migraine preventive therapy which may have affected BP during the 12 months of anti-CGRP treatment. Patients requiring adjustments to their antihypertensive therapy during the follow-up were not excluded from the analysis.

### Endpoints and assessments

The primary endpoint of the study was to assess whether treatment with anti-CGRP mAbs significantly affected systolic and/or diastolic BP during the 12-month follow-up from (that is BP ≥ 140 mmHg and/or diastolic BP ≥ 90 mmHg at any time during follow-up or patients with an increase in systolic blood pressure ≥ 20 mmHg and/or an increase in diastolic BP ≥ 10 mmHg at any time during follow-up).

The secondary endpoints were: (i) to assess any significant variation in systolic and diastolic blood pressure at any time point within any patient (ii) to evaluate the number of patients considered “Newly or Worsened Hypertensive” (i.e., a single measurement of > 140 mmHg for systolic, > 90 mmHg for diastolic at three and/or twelve months, and/or a new anti-hypertensive drug need); (iii) to evaluate baseline features related to newly hypertensive patients”; (iv) to assess whether treatment with anti-CGRP mAbs affected systolic and/or diastolic BP at 3-month follow-up from baseline (as for the same criteria above mentioned).

### Statistics

The sample size was based on the available population. Descriptive statistics were expressed as mean and standard deviation for continuous variables. We designed a linear mixed model (LMM) to assess the effect of Anti-CGRP(r) monoclonal antibodies on both systolic and diastolic pressure during 12 months. Time (three time points) and Anti-CGRP(r) monoclonal antibodies (erenumab, fremanezumab, and galcanezumab) were set up as fixed effects and single patients’ variability as a random effect. The Bonferroni’s correction was applied to account for multiple comparisons. The model fit was assessed using the Akaike information criterion (AIC) and Bayesian information criterion (BIC). The significance of fixed effects was assessed using the Wald chi-square test and the p-values. Results of the LMM were reported as estimated effects with marginal means, 95% confidence intervals (CIs), and p-values.

A binary logistic regression model was constructed to assess the association between “Newly Hypertensive Patients” as a categorial variable, and a set of covariates including age, sex, type of anti-CGRP(r) monoclonal antibodies, hypertension at baseline, diabetes, dyslipidemia, other cardiovascular comorbidities, and anti-hypertensive therapy at baseline. The binary logistic regression model fit was assessed with the Hosmer–Lemeshow test (chi-square = 7.55, df = 8, *p* = 0.478). Newly Hypertensive Patients are described in detail. The sample size was based on the available population. Post-Hoc statistical power analysis for the Linear Mixed Model assessed a > 80% power for the model. All statistical tests were two-tailed, and the p-values < 0.05 were considered statistically significant. Missing values were not imputed. Data were analyzed using SPSS version 24 (IBM Corp., Armonk, NY, USA).

### Standard protocol approvals, registrations, and patient consents

The study was approved by an independent ethics committee or local institutional review board at each participating site (protocol numbers: 20073 for Bologna, 50/2020/OSS/AOUMO and 625/2020/OSS/AUMO for Modena, and 30/20 OSS ComEt CBM for Rome Campus Biomedico). Written informed consent was obtained from all enrolled patients, both for study participation and data publication. All procedures were conducted according to the latest version of the Declaration of Helsinki.

## Results

### Descriptive results

During the recruitment period, a total of 155 patients (119 females, 36 males) met the inclusion criteria for the study. The mean age of our population was 64 years (± 3.1), with a range of 60 to 80 years and a clear predominance of females (76.7%). Forty patients were treated with Erenumab (mean age 62.6 years ± 2.0), 68 with Fremanezumab (mean age 67.4 ± 6.7), and 47 with Galcanezumab (mean age 63.0 ± 2.5). At the start of the study, 61 out of 155 patients (39.3%) had a known diagnosis of arterial hypertension at baseline and 65 (41.9% of the whole sample) had an ongoing antihypertensive therapy; four of those 65 patients had an anti-hypertensive medication for other medical reasons (three patients had a beta-blocker for tachycardia and one patient had candesartan as migraine prophylaxis). Demographics and historical data are presented in Table 1. At baseline, 13 of 155 patients (8.3%) had a systolic and/or diastolic value equal to or greater than 140/90 mmHg (maximum value was 145/95 mmHg) and were referred to their GP for monitoring or anti-hypertensive therapy prescription or adjustment; of these 13 patients, eight were known for hypertension and had ongoing specific therapy, whereas the remaining five patients had no history of hypertension.

**Table 1:** Baseline characteristics

		Total	Erenumab	Fremanezumab	Galcanezumab	P value
Sample	*N (%)*	155	40 (25.8)	68 (43.8)	47 (30.3)	
Age, *y*	*mean* ± *SD*	64 ± 3.1	62.6 ± 2.0	67.4 ± 6.7	63.0 ± 2.5	** < 0.001**
Sex						
Males	*N (%)*	36 (23.2)	10 (25)	10 (14.7)	16 (34.1)	0.052
Females	*N (%)*	119 (76.7)	30 (75)	58 (85.3)	31 (65.9)	
Cardiovascular Comorbidities						
Know Hypertension history	*N (%)*	61 (39.6)	16 (40.0)	27 (39.7)	18 (39.1)	0.996
Diabetes	*N (%)*	4 (2.6)	0 (0.0)	1 (1.4)	3 (2.1)	0.820
Dyslipidaemia	*N (%)*	52 (33.6)	13 (32.5)	24 (35.2)	15 (32.6)	0.938
Baseline Hypertension	*N (%)*	13 (8.3)	3 (7.5)	6 (8.8)	4 (8.5)	0.971
Anti-Hypertensive therapy	*N (%)*	65 (41.5)	16 (40)	29 (42.6)	20 (44.4)	0.897
Headache						
Episodic Migraine	*N (%)*	47 (31.0)	9 (22.5)	19 (27.9)	19 (43.1)	0.095
Chronic Migraine	*N (%)*	105 (69.0)	31 (77.5)	49 (72.1)	25 (56.9)	

*N* total number, *SD* Standard Deviation

**Bold an underlined font** indicates statistically significant values

For BP measurements availability: at baseline 100% of data were available; at three months 3/155 (1.9%) patients had missing values and after 12 months 10/155 (6.5%) measurements were missing, in both cases due to follow-up loss. Missing values were not imputed.

#### Systolic blood pressure

The mean systolic blood pressure (SBP) of the entire study population at baseline was 123.72 mmHg (95% CI, 120.83 – 126.60). At three and twelve months, the observed changes were + 0.44 (95% CI, -3.05 – + 3.95) and -0.315 (95% CI, -3.86 – 3.23), respectively. The estimated fixed effects showed an increase of + 0.34 mmHg (95% CI, -4.88 – 5.57, *p* = 0.897) at three months, with no significant effect after twelve months.

In the erenumab group, the changes in SBP at three and twelve months were 123.69 mmHg (95% CI, 119.08 – 128.30) and 123.99 mmHg (95% CI, 119.36 – 128.62), respectively, when compared to the baseline value of 123.74 (95% CI, 119.17 – 128.30). The estimated fixed effect was a decrease of -0.69 mmHg at three months (95% CI, -8.32—+ 6.94, *p* = 0.859) and no effect at twelve months.

In the fremanezumab group, the mean SBP at three and twelve months were 121.13 mmHg (95% CI, 117.78 – 124.47) and 123.42 mmHg (95% CI, 120.02 – 126.82), respectively, as compared to the baseline value of 125.13 (95% CI, 121.82 – 128.47). We observed an estimated fixed effect of -2.63 mmHg at three months (95% CI, -9,39—+ 4,12, *p* = 0.444) and no effect at twelve months.

For the galcanezumab group, the baseline mean SBP was 122.84 mmHg (95% CI, 118.12 – 126.44). At three and twelve months, the SBP values were 125.02 mmHg (95% CI, 120.79 – 129.28) and 124.69 mmHg (95% CI, 120.39 – 128.92), respectively. The estimated fixed effect at three months was + 0.12 mmHg (95% CI, -6.56 – 6.80, *p* = 0.917) with no effect noted after 12 months.

#### Diastolic blood pressure

The mean diastolic blood pressure (DBP) at baseline for the entire study population was 81.24 mmHg (95% CI, 78.32 – 83.24). The variations observed after three and twelve months were -1.19 (95% CI, -3,51 – 1.13) and -0.539 (95% CI, -2.84—1.81), respectively. The estimated fixed effects indicated an increase of + 1.24 mmHg (95% CI, -2.24 – 4.68, *p* = 0.490) at three months, with no significant effect thereafter. In patients treated with erenumab the mean DBP at three and twelve months were 78.96 mmHg (95% CI, 75.87 – 82.04) and 81.41 mmHg (95% CI, 78.29 – 84.54) respectively, compared to the baseline value of 81.78 (95% CI, 78.69 –84.86). We observed an estimated fixed effect of -3.67 mmHg at three months (95% CI, -8.73 – 1.38, *p* = 0.154) and an estimated zero effect at twelve months.

In the fremanezumab group, the mean DBP at three and twelve months were 78.89 mmHg (95% CI, 76.64 – 81.15) and 79.62 mmHg (95% CI, 77.34 – 81.91) respectively, compared to the baseline value of 80.55 (95% CI, 78.30 – 82.79). We observed an estimated fixed effect of -1.94 mmHg at three months (95% CI, -6.43—2.53, *p* = 0.394) and an estimated zero effect at twelve months.

The mean baseline DBP in the galcanezumab group was 81.51 mmHg (95% CI, 78.71 – 84–32). After three and twelve months DBP values were 82.41 mmHg (95% CI, 79.54 – 85.27) and 81.19 mmHg (95% CI, 78.29 – 84.09), respectively. After three months the estimated fixed effect of galcanezumab on diastolic pressure was 3.67 mmHg (95% CI, -5.07 – 4.99, *p* = 0.147), whereas no estimated effect was observed after 1 year.

Regarding the whole analysis, no statistically significant estimated effect was observed.

#### Newly or worsened hypertensive patients

During the study period, 20 out of 155 patients (12.9%) were considered Newly or Worsened Hypertensive. This group of patients had a mean age of 66.9 years (SD ± 4.88) and a females predominance (6 M, 14 F). Six patients were treated with Galcanezumab, 2 with erenumab, and 13 with fremanezumab.

### Patients with a pre-existing diagnosis of hypertension

Out of the 20 patients, 11 had previously been diagnosed with hypertension and were already receiving antihypertensive therapy. Among these eleven patients, five required either a higher dose or an additional medication to control hypertension. After twelve months, three patients' blood pressure readings were at the higher end of the normal range, whereas the remaining eight exhibited normal blood pressure values. When assessing baseline cardiovascular risk factors, it was noted that dyslipidemia was present in five of these 11 patients.

### Patients without a pre-existing diagnosis of hypertension

Of the remaining nine patients without a prior hypertension diagnosis, four (representing 2.5% of the total 155 participants) needed new antihypertensive therapy within the year-long follow-up. These four patients displayed no significant gender-based differences. Regarding cardiovascular risk factors, one patient was dyslipidemic, another was diagnosed with type 2 diabetes and dyslipidemia, and the rest exhibited no additional risk factors. The other five patients experienced a single instance of elevated blood pressure during the study, which, after careful monitoring in primary care, did not necessitate new medication.

### Baseline features

Upon comparing the baseline characteristics of patients who developed new or worsening hypertension to those whose blood pressure remained stable without the need for additional antihypertensive treatment, it was found that the only significant predictor of increased blood pressure was a higher baseline blood pressure reading (*p* = 0.002; Odds Ratio [OR] 8.83). For a comprehensive analysis of these baseline characteristics, please refer to Table 2.

**Table 2:** Newly or Worsened Hypertensive Patients

		Not-NWHP	NWHP	Odds Ratio (95% CI)	*p* value*	*P* value°
Sample	*N (%)*	135/155 (87)	20/155 (13)			
Age, *y*	*mean* ± *SD*	64.5 ± 5.3	66.9 ± 4.8	1.06 (0.92 – 1.21)	0.07	**-**
Sex (Females)	*N (%)*	*105/135 (77)*	*14/20 (70)*	*1.70 (0.51 – 5.61)*	*0.46*	*-*
Cardiovascular Comorbidities						
Know Hypertension history	*N (%)*	52/135 (38)	9/20 (45)	0.11 (0.009 – 1.32)	0.61	-
Diabetes	*N (%)*	3/135 (2.2)	1/20 (5)	2.28 (0.16 – 30.82)	0.47	-
Dyslipidaemia	*N (%)*	45/135 (33)	7/20 (35)	0.70 (0.19 – 2.52)	0.86	-
Baseline Hypertension	*N (%)*	7/135 (5)	7/20 (35)	**8.83 (2.29 – 34.02)**	** < 0.001**	**0.002**
Anti-Hypertensive therapy	*N (%)*	55/135 (40)	11/20 (42)	4.41 (0.52 – 32.93)	0.25	-
Monoclonal Antibodies anti-CGRP(r)						
Erenumab	*N (%)*	38/135 (28)	2/20 (10)	0.47 (0.08 – 2.83)	0.17	-
Fremanezumab	*N (%)*	56/135 (41)	12/20 (60)	2.93 (0.45 – 18.96)	0.13	-
Galcanezumab	*N (%)*	41/135 (30)	6/20 (30)	2.08 (0.35- 12.36)	0.95	-

*N* Number, *SD *Standard Deviation, *Not-NWHP* Not Newly or Worsened Hypertensive Patients, *NWHP* Newly or Worsened Hypertensive Patients

^*^ = *p* values comparing baseline features

° = *p* values considering the logistic binary regression equation

**Bold and underlined font** indicates statistically significant values

## Discussion

This study is the first to prospectively monitor blood pressure in a population of episodic and chronic migraine patients aged 60 and over. The study participants included individuals with cardiovascular risk factors such as hypertension, dyslipidemia, and diabetes who received anti-CGRP(r) monoclonal antibodies for a period of one year. Blood pressure was measured at the start of treatment, and then again at three and 12 months, with cardiovascular events and changes in pharmacotherapy recorded.

The study was conducted with the expectation that a high percentage of the cohort would have hypertension (42% of our cohort), given their age group, and 39% were already on antihypertensive therapy. However, only a small fraction of the participants had blood pressure readings slightly above normal threshold at the beginning of the study, and they were still treated with anti-CGRP(r) mAbs with caution advised for blood pressure monitoring.

Throughout the study, no cardiovascular events and no significant fluctuations in blood pressure were observed. To ensure the study captured a realistic clinical setting, we included in the analysis patients who required adjustments to their antihypertensive therapy during follow-up. This decision was based on our consideration that such adjustments could indicate worsening hypertension, which is a critical outcome to monitor. By including these patients, we aimed to provide a more comprehensive view of the potential effects of anti-CGRP monoclonal antibodies on blood pressure.

The estimated effects of the three anti-CGRP(r) monoclonal antibodies on both systolic and diastolic blood pressure were negligible at every time point. These findings align with data from large-scale randomized clinical trials and real-world data from early studies that found no evidence of cardiovascular risks in migraine patients. [18, 19, 22–25, 27, 32–34]

Our research is significant in that it exclusively focused on patients over 60 years of age, a population at a greater risk for hypertension and cardiovascular events. Although a recent study by de Vries Lentsch et al. reported a small increase in blood pressure among patients with migraine treated with anti-CGRP mAbs, it failed to show a significant increase in the incidence of hypertension[29]. Nevertheless, this finding prompted a discussion on the potential for hypertension risk in the migraine treatment guidelines. Our results suggest that CGRP-mediated vasodilation may serve as an emergency response mechanism rather than a regulator of resting blood pressure levels [12]. However, these findings are currently limited to animal models and have not been confirmed in human studies. Furthermore, a recent study on CGRP plasma levels in patients with acute ischemic stroke found no change in CGRP levels from the day of admission to 24 h later, and no link between CGRP levels and clinical outcomes or brain imaging findings. These findings highlight the complexity of CGRP's role in cardiovascular events and underscore the need for further research in human subjects to clarify these relationships. [35, 36]

In terms of "Newly or Worsened Hypertensive patients" (i.e., a single measurement of > 140 mmHg for systolic, 90 mmHg for diastolic, and/or a new anti-hypertensive drug need), we observed a proportion of 20 out of 155 patients (12.9%) over a one-year period. However, upon closer examination of these data, we found that 11 of the 20 NWHP had a history of hypertension, representing a higher preexisting CV risk group. Moreover, less than half of these patients required adjustment for antihypertensive therapy, and by the end of the follow-up period, more than 70% of these patients had normal blood pressure values. Among the 9 patients without a history of hypertension, 5 showed elevated blood pressure at only one-time point, and home monitoring did not show consistently elevated blood pressure values and did not require the initiation of pharmacological therapy, potentially suggesting a white coat effect. The remaining patients without a history of hypertension required initiation of antihypertensive therapy in only four cases, that is, 2.5% of the total group of 155 patients. Although we cannot compare these data with a control group, the numbers appear to be in line, if not even lower, than the consolidated annual incidence of hypertension in the over 50 s age group and significantly lower than the incidence in the over 65 s age group. [37, 38] Among the baseline predictors of new or worsened hypertension, only an altered blood pressure value at the baseline visit was significant, further indicating that risk factors were pre-existing to anti-CGRP therapy. Notably, a larger proportion of patients with new or worsened hypertension were observed in the fremanezumab group, which could be related to the higher average age in this subgroup, increasing the likelihood of hypertension development or exacerbation. These observations could be in line with recent evidence from a large retrospective study that observed a higher number of antihypertensive medications during treatment with anti-CGRP mAbs only in patients with pre-existing hypertension. [39]

Although monoclonal antibodies targeting the CGRP pathway can act differently depending on whether they target the ligand or receptor, our study was not designed to compare different mAbs. However, we did not observe any significant differences in blood pressure values throughout the study period.

The strengths of our research include the prospective data collection, extended follow-up period, and minimal data loss, and it is the first real-world assessment of anti-CGRP(r) mAb treatment in the over-60 migraine patient demographics.

Nonetheless, it is imperative to acknowledge certain limitations, as our study did not include a control group of migraine patients over 60 years of age who were not treated with anti-CGRP monoclonal antibodies. Given the relatively low prevalence of this condition (migraine patients over 60 years of age who received anti-CGRP(r) mAb treatment), our study was designed based on the available population. Consequently, we cannot guarantee the generalizability of our findings. Furthermore, we must consider the potential for selection bias as we excluded patients aged > 60 years with major known cardiovascular events, which may have led to a population with a lower risk of developing hypertension. Additionally, it is important to consider the limitations of a single blood pressure measurement in an outpatient clinic, which may result in false negatives and cannot provide a comprehensive picture of the blood pressure state of every patient. On the other hand, most hypertension diagnoses in primary care and antihypertensive therapy prescriptions are based on blood pressure measurement in outpatient clinics, and the white coat effect could result in a false positive elevated BP, potentially leading to higher values than those observed in a home monitoring condition. Therefore, extended and continuous blood pressure monitoring would offer a more accurate assessment of the true arterial pressure in patients treated with anti-CGRP(r) mAbs. Moreover, with the increasing use of these drugs, vigilant and comprehensive monitoring is essential to detect any potential side effects, particularly in patients aged > 60 years with a higher risk of comorbidities. It is also crucial to investigate the potential role of CGRP(r) blockade in the acute phase of cardiovascular or cerebrovascular events, in terms of vascular collateral efficiency and functional outcomes.

## Conclusion

Our findings suggest that monoclonal antibodies targeting the CGRP pathway, specifically erenumab, fremanezumab, and galcanezumab, did not result in blood pressure changes over a one-year period, even among individuals aged over 60 years. Moreover, the incidence of new or worsening cases of hypertension did not exceed what is typically observed in population-based studies. Notably, elevated baseline BP was the sole predictor of new or exacerbated hypertension.

While our study was not expressly designed to compare treatments, no discernible differences in blood pressure outcomes were detected among the three anti-CGRP(r) options, suggesting that they are safe for migraine prevention in older adults, even those with vascular risk factors.

Nevertheless, ongoing vigilance is essential for monitoring long-term cardiovascular effects and assessing functional outcomes following acute cardiovascular or cerebrovascular events in patients receiving anti-CGRP therapy.

## Key points

- We evaluated blood pressure trends in a population of elderly migraineurs undergoing an anti-CGRP mAbs and we did not observe any significant change in blood pressure over a one-year period.
- Considering the new o worsened hypertensive patients we found a slightly lower incidence than hypertension incidence derived from age matched population studies.
- The only baseline factor associated with new or worsened hypertension was a baseline high pressure value, further assessing the pre-existing nature of the cardiovascular risk.

## Data Availability

Complete anonymized dataset is available upon reasonable request to the corresponding author.

## Declarations

### Standard protocol approvals, registrations, and patient consents

The study was approved by an independent ethics committee or local institutional review board at each participating site (protocol numbers: 20073, 50/2020/OSS/AOUMO and 625/2020/OSS/AUMO, and 30/20 OSS ComEt CBM, for the three participant centres). Written informed consent was obtained from all enrolled patients, both for study participation and data publication. All procedures were conducted according to the latest version of the Declaration of Helsinki.
